# Bee Products Prevent Agrichemical-Induced Oxidative Damage in Fish

**DOI:** 10.1371/journal.pone.0074499

**Published:** 2013-10-03

**Authors:** Daiane Ferreira, Helio Carlos Rocha, Luiz Carlos Kreutz, Vania Lucia Loro, Alessandra Marqueze, Gessi Koakoski, João Gabriel Santos da Rosa, Darlan Gusso, Thiago Acosta Oliveira, Murilo Sander de Abreu, Leonardo José Gil Barcellos

**Affiliations:** 1 Programa de Pós-Graduação em Farmacologia, Universidade Federal de Santa Maria (UFSM), Santa Maria, Rio Grande do Sul, Brazil; 2 Programa de Pós-Graduação em Bioexperimentação, Universidade de Passo Fundo (UPF), Passo Fundo, Rio Grande do Sul, Brazil; 3 Programa de Pós-Graduação em Bioquímica Toxicológica, Universidade Federal de Santa Maria (UFSM), Santa Maria, Rio Grande do Sul, Brazil; 4 Programa de Pós-Graduação em Avaliação de Impactos Ambientais, Centro Universitário La Salle (Unilasalle), Canoas, Rio Grande do Sul, Brazil; Catalan Institute for Water Research (ICRA), Spain

## Abstract

In southern South America and other parts of the world, aquaculture is an activity that complements agriculture. Small amounts of agrichemicals can reach aquaculture ponds, which results in numerous problems caused by oxidative stress in non-target organisms. Substances that can prevent or reverse agrichemical-induced oxidative damage may be used to combat these effects. This study includes four experiments. In each experiment, 96 mixed-sex, 6-month-old *Rhamdia quelen* (118±15 g) were distributed into eight experimental groups: a control group that was not exposed to contaminated water, three groups that were exposed to various concentrations of bee products, three groups that were exposed to various concentrations of bee products plus tebuconazole (TEB; Folicur 200 CE™) and a group that was exposed to 0.88 mg L^−1^ of TEB alone (corresponding to 16.6% of the 96-h LC_50_). We show that waterborne bee products, including royal jelly (RJ), honey (H), bee pollen (BP) and propolis (P), reversed the oxidative damage caused by exposure to TEB. These effects were likely caused by the high polyphenol contents of these bee-derived compounds. The most likely mechanism of action for the protective effects of bee products against tissue oxidation and the resultant damage is that the enzymatic activities of superoxide dismutase (SOD), catalase (CAT) and glutathione-S-transferase (GST) are increased.

## Introduction

In southern South America and other parts of the world, aquaculture is still considered a complementary activity to agriculture. In agricultural, fish cultures are often located in close proximity to agricultural areas and are fed by water from springs that run through cultivated soil. Consequently, small amounts of agrichemicals can reach aquaculture ponds [1], leading to the exposure of non-target organisms to numerous problems, such as oxidative stress.

Oxidative stress refers to a disturbance in the balance between oxidants and antioxidants, which favors an abundance of oxidants [2]. To maintain oxidative balance, organisms utilize an enzymatic antioxidant defense system that is comprised of enzymes, such as superoxide dismutase (SOD), catalase (CAT) and glutathione-S-transferase (GST), as well as a nonenzymatic defense system comprised of ascorbic acid (AsA), total thiols (TT) and other components [2], [3]. When these systems fail to maintain the oxidative balance, pro-oxidants may induce higher lipid peroxidation (LPO) ratios and cause damage to tissues and cells [4].

Most agricultural models utilize agrichemicals intensely during crop production; therefore, substances that can prevent or reverse agrichemical-induced oxidative damage are beneficial. Bee products are a good option because they are important sources of polyphenols [5]–[9], which are known to be antioxidants and free radical scavengers.

Recently, the antioxidant properties of natural products have been intensively researched to find economically viable substances with low environmental impact. Antioxidants inhibit the reactive oxygen species (ROS) generated by cellular metabolism, which has fueled the analysis of various antioxidant compounds as isolated products and *in natura* [10].

In a previous study, we found that honey (H) and bee pollen (BP) reversed agrichemical-induced oxidative stress, whereas the alcoholic extract of propolis (P) induced a protracted oxidative reaction [11]. In that study, we used bee products at a unique arbitrary concentration and did not test royal jelly (RJ).

In this study, we investigated different waterborne concentrations of H, BP, water extract of P and RJ as protectants against the damaging effects caused by exposing fish to the fungicide tebuconazole (TEB).

## Materials and Methods

In the following experiments, which were repeated for each bee product, we assayed three concentrations of each product to determine the lowest active concentration. The experiments were conducted in February of 2012 in the facilities of the University of Passo Fundo (28°15′S/52°24″W, 687 m above sea level).

### Ethical Note

This study was approved by the Ethics Commission for Animal Use (CEUA) of the Universidade de Passo Fundo, UPF, Passo Fundo, RS, Brazil (Protocol#3/2011-CEUA, July 2009) and has met the guidelines of the Brazilian College for Animal Experimentation (COBEA; http://www.cobea.org.br).

### Fish and housing conditions

We used the South American catfish jundiá (*Rhamdia quelen*, Heptapteridae, Teleostei) as a model fish, which are usually cultured in ponds near crop production fields. This species is endemic to southern South America and suitable for cultivation in any region with a temperate or subtropical climate. The toxicological responses of this species have been extensively researched [12]–[16].

Three hundred eighty four (96 fish per experiment) mixed-sex, 6-month-old *R. quelen* weighing 118±15 g were bred and maintained at the Universidade de Passo Fundo. The fish inhabited a 6200-L plastic tank before being transferred to the experimental tanks where they were maintained under a natural photoperiod. The fish were fed twice daily (10:00 and 16:00 h) with commercially extruded food at 5% of their body weight (42% crude protein, 3400 kcal kg^−1^ of digestive energy (DE)). The water temperature (25±1°C) and dissolved oxygen concentrations (5.9 mg L^−1^) were measured with a YSI model 550A oxygen meter (Yellow Spring Instruments, Yellow Spring, USA). The pH values (6.4–7.2) (Bernauer pH meter), total ammonia-N (<0.6 mg L^−1^), total alkalinity (55 mg L^−1^ CaCO_3_) and hardness (60 mg L^−1^ CaCO_3_) of the water were measured using colorimetric tests.

### Experimental design

For each experiment, the fish were distributed into eight experimental groups (24 1000-L fiberglass tanks, three tanks per treatment group) after acclimation. Each treatment group was divided into three tanks containing 200 L of chlorine-free, well-aerated tap water; no water changes or agrichemical replacement methods were enacted. Similar sex ratios were maintained between the replicates of the treatment groups. The fish housed in tanks 1 to 3 (the C group) were not exposed to any contaminants or bee products, whereas the fish housed in tanks 13 to 15 were exposed to 0.88 mg L^−1^ (corresponding to 16.6% of the 96-h LC_50;_ [13]) of TEB (Folicur 200 CE™).

TEB is a fungicide that is widely applied to cereal crops; this compound is a class II environmental hazard (dangerous to the environment and highly toxic to aquatic organisms) according to the Instituto Brasileiro do Meio Ambiente e dos Recursos Naturais Renováveis (IBAMA) and the Environmental Protection Agency (EPA). TEB exhibits systemic action and persists in the environment for 20–25 days. Folicur 200 CE, which is a commercial TEB formulation, is toxic to aquatic organisms and causes oxidative stress [16], [17] as well as endocrine disruption in fish [14].

TEB exhibits toxic effects on the liver, blood and adrenal glands. It is rapidly absorbed from the gastrointestinal tract and reaches peak plasma concentrations within hours. TEB metabolism in the body is affected mostly by oxidation and is eliminated from organs and tissues rapidly through fecal and urinary routes [18]. This compound demonstrates low bioaccumulation and the bioaccumulation factor is 65 (average) for intact fish (measured in several species of fish) [19]. Fish are particularly sensitive to the influence of pesticides because they absorb and retain dissolved xenobiotics in the water by active or passive transport [20].

In the RJ experiment, the fish housed in tanks 4 to 12 were exposed to waterborne RJ at three concentrations (three tanks per concentration): 0.005, 0.015 and 0.025 g L^−1^. The fish housed in tanks 16 to 24 were exposed to the same RJ concentrations in combination with TEB (RJ+TEB). In the second set of experiments, the fish were exposed to 0.025, 0.075 or 0.125 g L^−1^ of H as well as the same H concentrations in combination with TEB (H+TEB). In the third set of experiments, BP was added to the water at concentrations of 0.01, 0.03 and 0.05 g L^−1^ either alone (tanks 4 to 12) or in combination with TEB (BP+TEB; tanks 16 to 24). In the fourth set of experiments, we added 0.01, 0.05 and 0.1 g L^−1^ of aqueous extract of P to tanks 4 to 12. These three concentrations were administered in combination with 0.88 mg L^−1^ of TEB (P+TEB) in tanks 16 to 24.

In all experiments, the conditions (treatments) were applied for 96 hours (acute exposure) and all fish were subsequently sampled. The study followed the same protocol that is commonly used for acute toxicity studies resulting from a single exposure [13].

### Sampling

The fish were anesthetized with MS-222 in NaHCO_3_ (300 mg L^−1^) buffer, captured and killed by spinal section/decapitation before being dissected for tissue collection. The liver, kidney and brain tissues were immediately frozen in liquid nitrogen and stored for analysis.

After sampling, all of the dead fish were frozen and shipped to a biological waste facility. After each experiment, the contaminated water was retained in fiberglass tanks for a minimum of 30 days before being percolated in septic ponds. The tanks were cleaned with running water and rinsed with ethanol.

### Chemicals and honeybee products

TEB was obtained commercially. Bovine serum albumin, 5,5′-dithiobis-(2-nitrobenzoic acid) (DTNB), Triton X-100, hydrogen peroxide (H_2_O_2_), malondialdehyde (MDA), 2-thiobarbituric acid (TBA) and sodium dodecyl sulfate (SDS) were purchased from Sigma Chemical Co. (St. Louis, MO, USA). The H, P, RJ and BP used in this study were produced at the Centro de Pesquisa Agropecuária (CEPAGRO) at the Universidade de Passo Fundo, Brazil.

The concentration of TEB in the water was analyzed immediately after inoculation in addition to 48 and 96 hours after inoculation using high-pressure liquid chromatography (HPLC) with a TEB-specific method developed by Zhao [21]. The concentrations of all other compounds were analyzed using the method described by Zanella *et al*. [22].

### Parameters measured

The protein levels were estimated spectrophotometrically using the method described by Bradford [23], and bovine serum albumin was used as the standard.

The peroxides produced by the fish can be indirectly quantified using the TBARS assay, which measures a major product of LPO (malonic dialdehyde) [24]. This assay is performed by reacting MDA with TBA, and the results are assessed optically. The tissue samples were homogenized using 10% trichloroacetic acid (TCA) with a motor-driven Teflon pestle; the homogenates were centrifuged at 1000×*g* for 10 min. The liver homogenates (100–400 µL) were added to 8.1% sodium dodecyl sulfate (SDS), 2.5 M acetic acid (pH 3.4) and 0.8% thiobarbituric acid, and the final volume was adjusted to 2.0 mL. The reaction mixture was placed in a microcentrifuge tube and incubated for 90 min at 95°C. After cooling, the sample was centrifuged at 5000×*g* for 10 min, and the optical density was determined at 532 nm. The TBARS levels are expressed as nmol MDA per mg of protein, as described by Ohkawa *et al*. [25].

For protein carbonyl determination, liver tissue was homogenized in 10 volumes (w/v) of 10 mM Tris–HCl buffer (pH 7.4) with a glass homogenizer. The protein carbonyl content was determined using the method described by Yan *et al*. [26] with minor modifications. Briefly, the homogenates were diluted to 0.7–0.8 mg mL^−1^ of protein, and 1 mL aliquots were mixed with 0.2 mL of 2,4-dinitrophenylhidrazine (10 mM DNPH) or 0.2 mL of 2 M HCl. After 1 h of incubation at room temperature in a dark room, 0.5 mL of denaturing buffer (150 mM sodium phosphate buffer (pH 6.8) containing 3% SDS), 2.0 mL of heptanes (99.5%) and 2.0 mL of ethanol (99.8%) were added sequentially, vortexed for 40 s and centrifuged for 15 min. The isolated protein was washed two times with 1 mL of ethyl acetate/ethanol (1∶1 v v^−1^) and suspended in 1 mL of denaturing buffer. Each DNPH sample was read at 370 nm in a Femto Scan spectrophotometer against a corresponding sample (blank), and the total carbonylation level was calculated using a molar extinction coefficient of 22000 M^−1^ cm^−1^.

### Enzymatic defense antioxidant system

Catalase (CAT; EC 1.11.1.6) activity was assayed using ultraviolet spectrophotometry [27]. Samples of liver tissue were homogenized in a Potter–Elvehjem glass/Teflon homogenizer with 20 mM potassium phosphate buffer (pH 7.4 with 0.1% Triton X-100 and 150 mM NaCl, 1∶20 dilution) and centrifuged at 10,000×*g* for 10 min at 4°C. Briefly, the assay mixture consisted of 2.0 mL potassium phosphate buffer (50 mM at pH 7.0), 0.05 mL H_2_O_2_ (0.3 M) and 0.05 mL homogenate. Changes in the H_2_O_2_ absorbance after 60 s were measured at 240 nm. The catalase activity was calculated as µmol mg^−1^ protein min^−1^.

Glutathione S-transferase (GST) activity was determined using the method described by Habig *et al*. [28]. The reaction mixture consisted of 33 mM Hepes buffer (pH 7.5), 1.5 mM GSH, 1.5 mM 1-chloro-2,-dinitrobenzene (CDNB) and water in a total volume of 1 mL. The conjugation of GSH with CDNB using GST activity was recorded spectrophotometrically at 340 nm over 3 min. The activity was reported as nmol of CDNB conjugate formed min^−1^ mg^−1^ of protein.

Superoxide dismutase (SOD) activity was performed in liver tissue; this method is based on the inhibition of the reaction of radical superoxide with adrenalin, as described by McCord and Fridovich [29]. In this method, the SOD present in the sample competes with the detection system for radical superoxide molecules. A unit of SOD is the amount of enzyme that inhibits the speed of oxidation of adrenalin by 50%. The oxidation of adrenalin generates a colored product, adrenochrome, which is detected using a spectrophotometer. SOD activity is determined by measuring the speed of adrenochrome formation, which is observed at 480 nm, in a reaction medium containing glycine-NaOH (50 mM at pH 10) and adrenalin (1 mM).

### Non-enzymatic anti-oxidants

The ascorbic acid (AsA) content of the brain and liver was determined at 524 nm. Briefly, brain and liver samples were homogenized (1∶10 w/v) in 20% TCA and centrifuged at 11,300×*g* for 3 min. Samples (250 µL) of the supernatant were mixed with 250 µL of water plus 25 µL 0.02% 2,6-dichlorophenolindophenol; the resultant mixture was incubated for 1 h at room temperature. After, 250 µL of a 2% thiourea and 5% metaphosphoric acid solution and 250 µL of 0.2% dinitrophenylhydrazine in 12 M sulfuric acid were added to the solution. The reaction tubes were incubated in a 60°C water bath for 3 h. After the incubation, 500 µL of 18 M sulfuric acid was added, and the tubes were centrifuged at 500×*g* for 10 min. The absorbance was read and compared to a standard containing 100 nmol of AsA; the results were expressed in µmol g^−1^ of tissue [30].

### Statistical analysis

The means ± standard error of the mean (SEM) for each group were calculated. Hartley's and Kolmogorov–Smirnov tests were used to determine the homogeneity of the variance and normality, respectively; the log-transformation was performed when necessary. Because the premises for using parametric tests were met, we conducted a multivariate analysis of the variance complemented by Tukey's multiple range test, which was used to compare all of the means using the GraphPad InStat 3.00 statistical package (GraphPad Software; San Diego, California, USA). Statistical significance was accepted at p<0.05.

## Results

Immediately after inoculation, the TEB concentration measured in the water was close to 0.88 mg L^−1^ (97.3%, 0.856 mg L^−1^), which was the nominal concentration. Forty-eight hours after inoculation, the TEB concentration reached 79% of the nominal concentration (0.695 mg L^−1^), and 96 hours after inoculation, the TEB concentration was approximately 50% of the nominal concentration (0.439 mg L^−1^). Exposure to TEB did not cause mortality in the fish.

### Royal jelly

**Figure 1:**
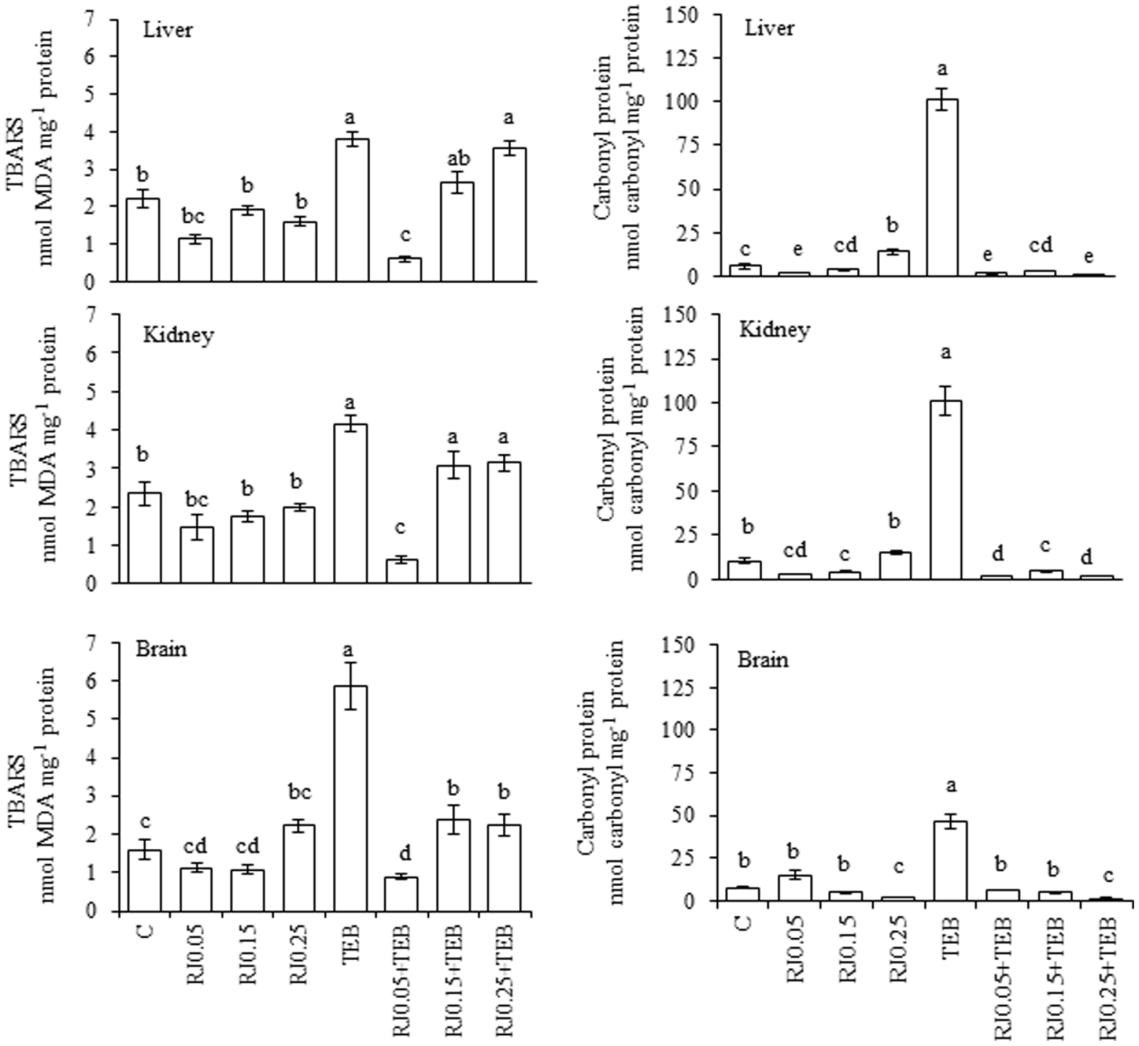
Levels of TBARS (nmol MDA mg^−1^ protein) and protein carbonyls (nmol carbonyl mg^−1^ protein) in *Rhamdia quelen* exposed to tebuconazole (TEB), royal jelly (RJ) or a combination of RJ and TEB. Contaminant-free (C). The different small letters indicate significant differences between the means (ANOVA followed by Tukey's multiple range test). The values represent the means ± SEM; n = 12. p<0.05.

**Figure 2:**
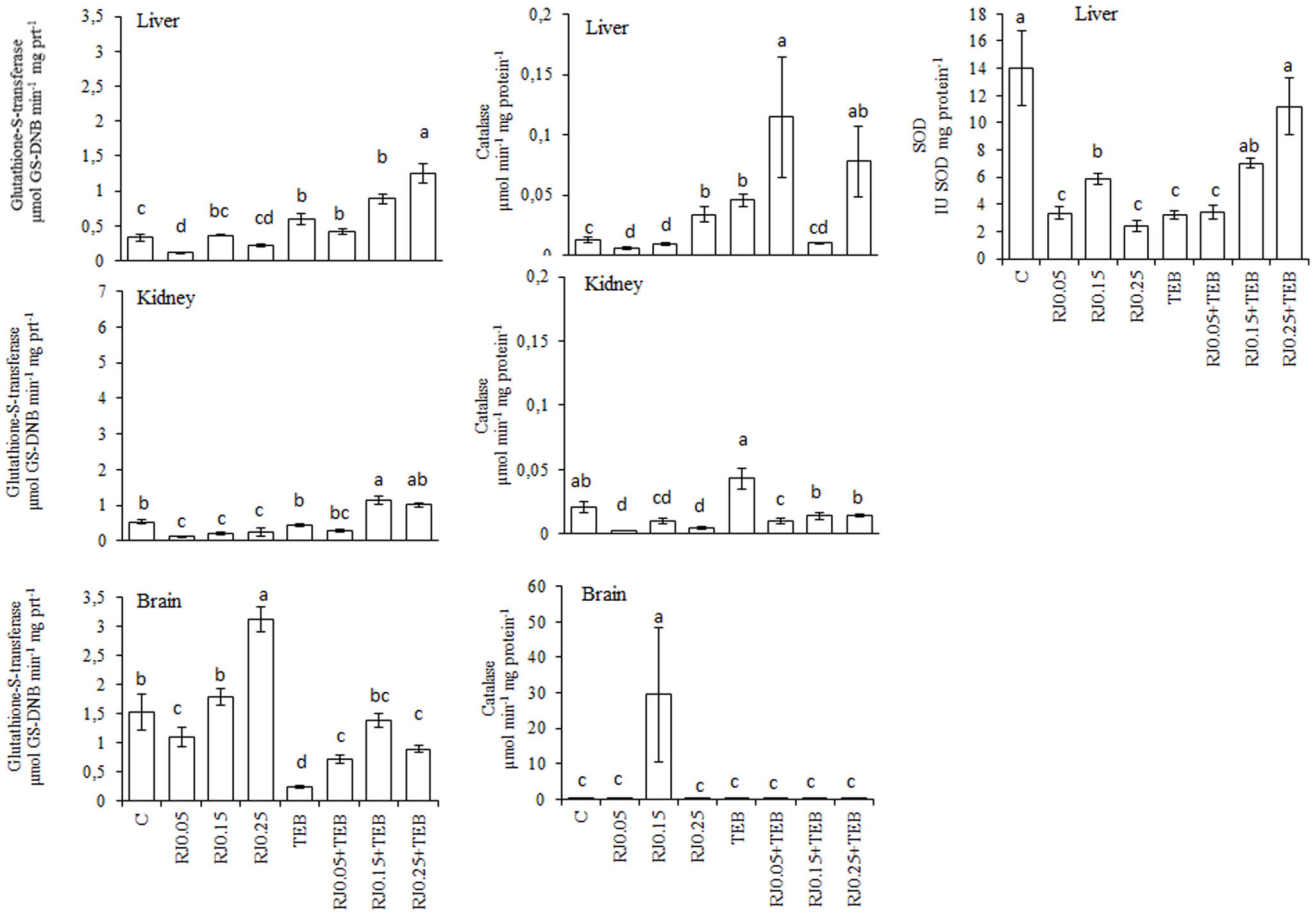
Levels of Glutathione-S-transferase (GST, µmol GS-DNB min^−1^ mg protein^−1^), catalase (CAT, µmol min^−1^ mg protein^−1^) and superoxide dismutase (SOD, IU SOD mg protein^−1^) activities in *Rhamdia quelen* exposed to tebuconazole (TEB), royal jelly (RJ) or a combination of RJ and TEB. Contaminant-free (C). The different small letters after the means indicate significant differences (ANOVA followed by Tukey's multiple range test); p<0.05, n = 12.

The TEB treatment increased the TBARS generation and protein carbonylation (Fig. 1). The lowest RJ concentration decreased TEB-induced TBARS generation in the liver and kidneys. In the brain, all three RJ concentrations reduced the TEB-induced TBARS generation. All of the RJ concentrations blocked the TEB-induced increases in protein carbonyls. The results for the enzyme activity assays are depicted in Fig. 2. The GST activity was increased in the liver for treatments using RJ0.015+TEB and RJ0.025+TEB; CAT activity was also increased with RJ0.005+TEB and RJ0.025+TEB, whereas SOD was augmented by RJ0.015+TEB and RJ0.025+TEB. In the kidneys, the GST activity increased under RJ0.015+TEB and RJ0.025+TEB treatments, whereas the CAT activity was similar to the control values. In the brain, the GST and CAT activities were similar to the control values at all three RJ+TEB concentrations.

### Honey

**Figure 3:**
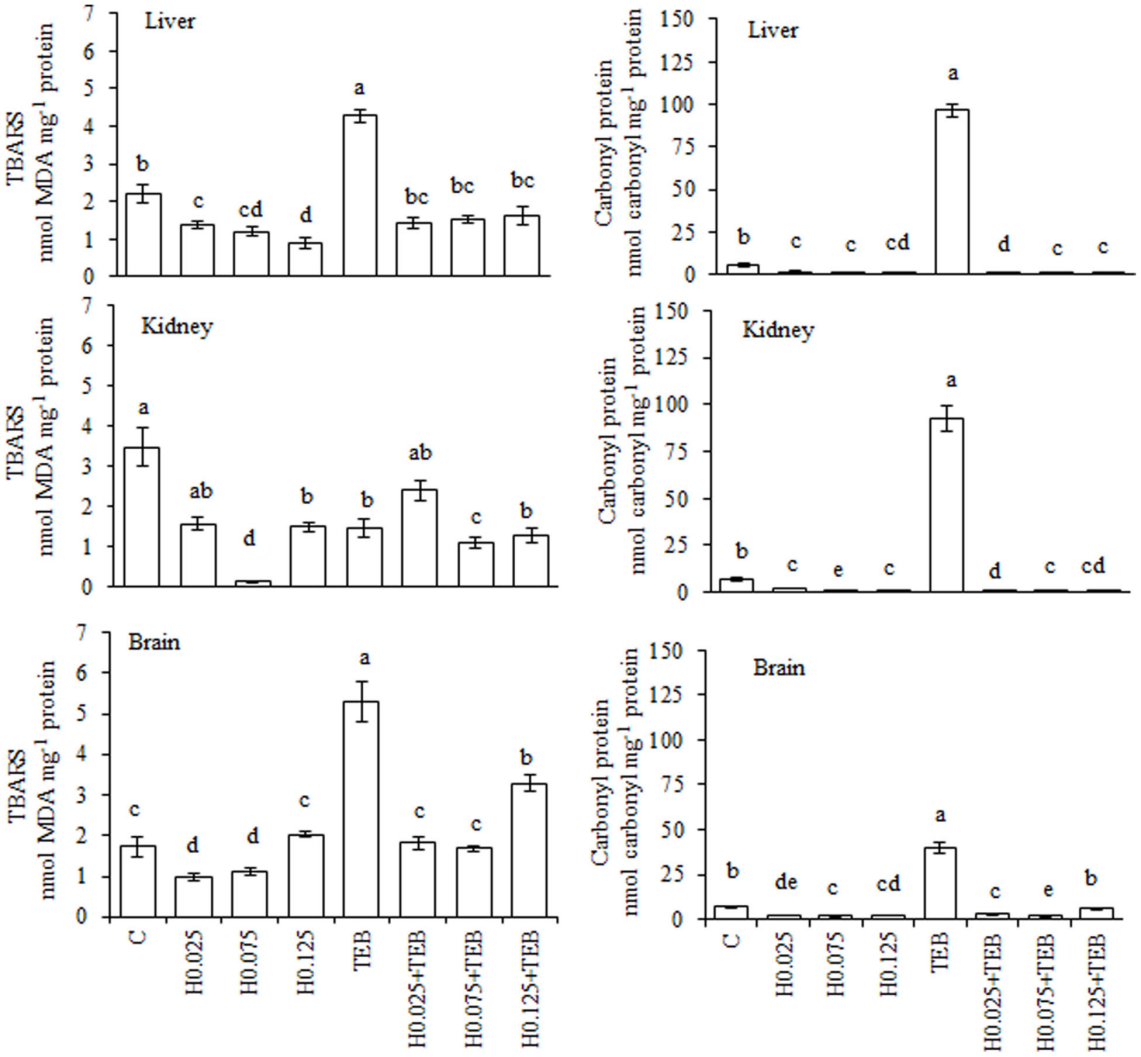
Levels of TBARS (nmol MDA mg^−1^ protein) and protein carbonyls (nmol carbonyl mg^−1^ protein) in *Rhamdia quelen* exposed to tebuconazole (TEB), honey (H) or a combination of H and TEB. Contaminant-free (C). The different small letters indicate significant differences between the means (ANOVA followed by Tukey's multiple range test). The values represent the means ± SEM; n = 12. p<0.05.

**Figure 4:**
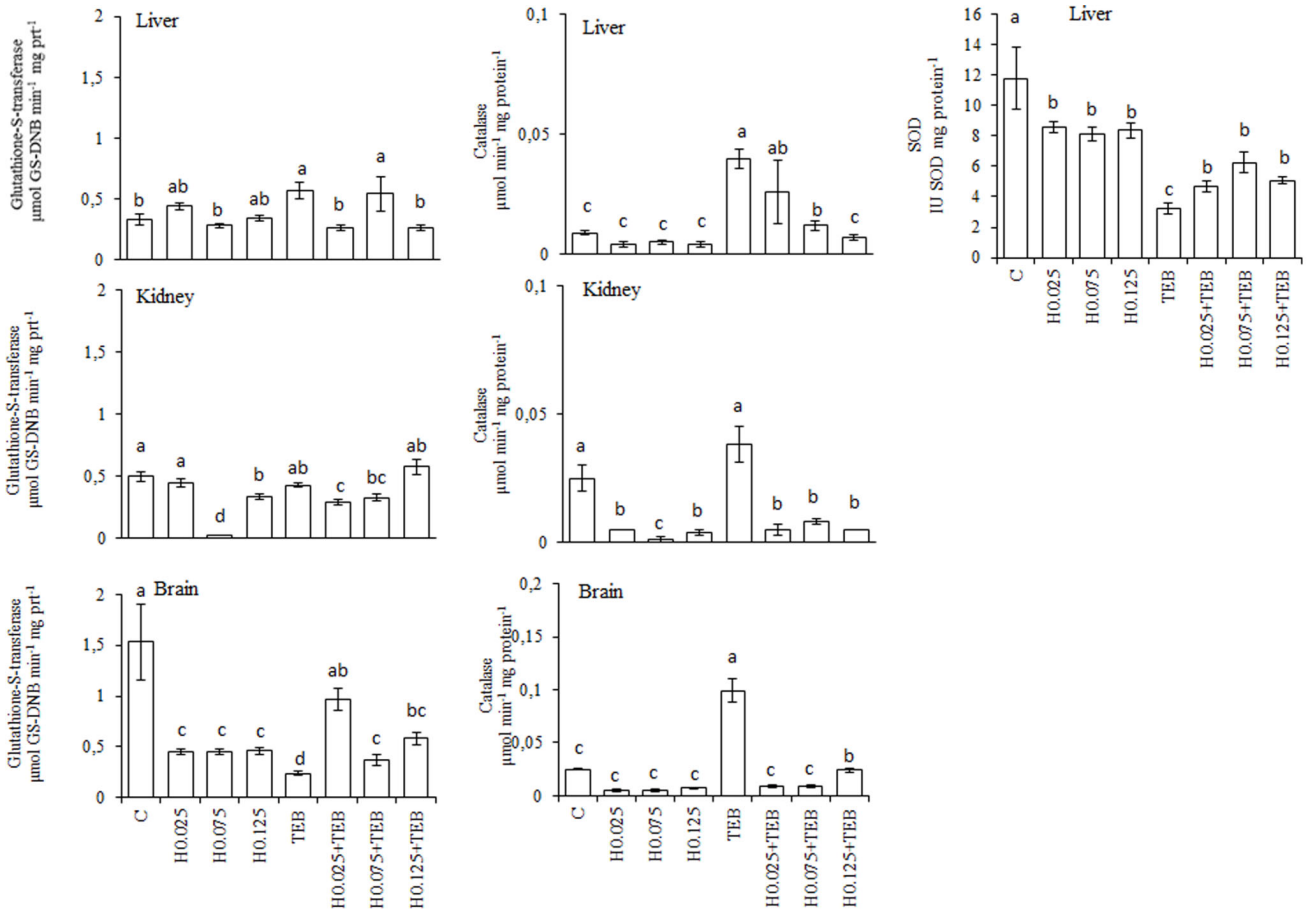
Levels of Glutathione-S-transferase (GST, µmol GS-DNB min^−1^ mg protein^−1^), catalase (CAT, µmol min^−1^ mg protein^−1^) and superoxide dismutase (SOD, IU SOD mg protein^−1^) activities in *Rhamdia quelen* exposed to tebuconazole (TEB), honey (H) or a combination of H and TEB. Contaminant-free (C). The different small letters after the means indicate significant differences (ANOVA followed by Tukey's multiple range test); p<0.05, n = 12.

Treatment with TEB increased the TBARS generation and protein carbonylation in all examined tissues (Fig. 3) except for the kidneys. In the liver and brain tissues, H completely blocked the TEB-induced TBARS generation; however, only the highest two H concentrations exhibited this effect in the kidneys. All of the concentrations of H blocked TEB-induced protein carbonylation in all tissue samples. In Fig. 4, we present the results of all enzymatic activity assays. In the liver, the GST activity increased under H0.075+TEB treatment, CAT increased with H0.025+TEB and H0.075+TEB and was augmented in TEB-exposed fish and SOD activity was increased under all three H+TEB treatments relative to the control values. In the kidney tissue, GST increased in the H0.125+TEB group, whereas all H+TEB were similar to the control group for CAT activity during H treatments. Regarding the brain tissue, GST activity was increased for the three H+TEB treatments compared to the TEB-exposed fish, whereas CAT activity was similar to the control in all three H+TEB groups and was augmented in the TEB-exposed fish.

### Propolis

**Figure 5:**
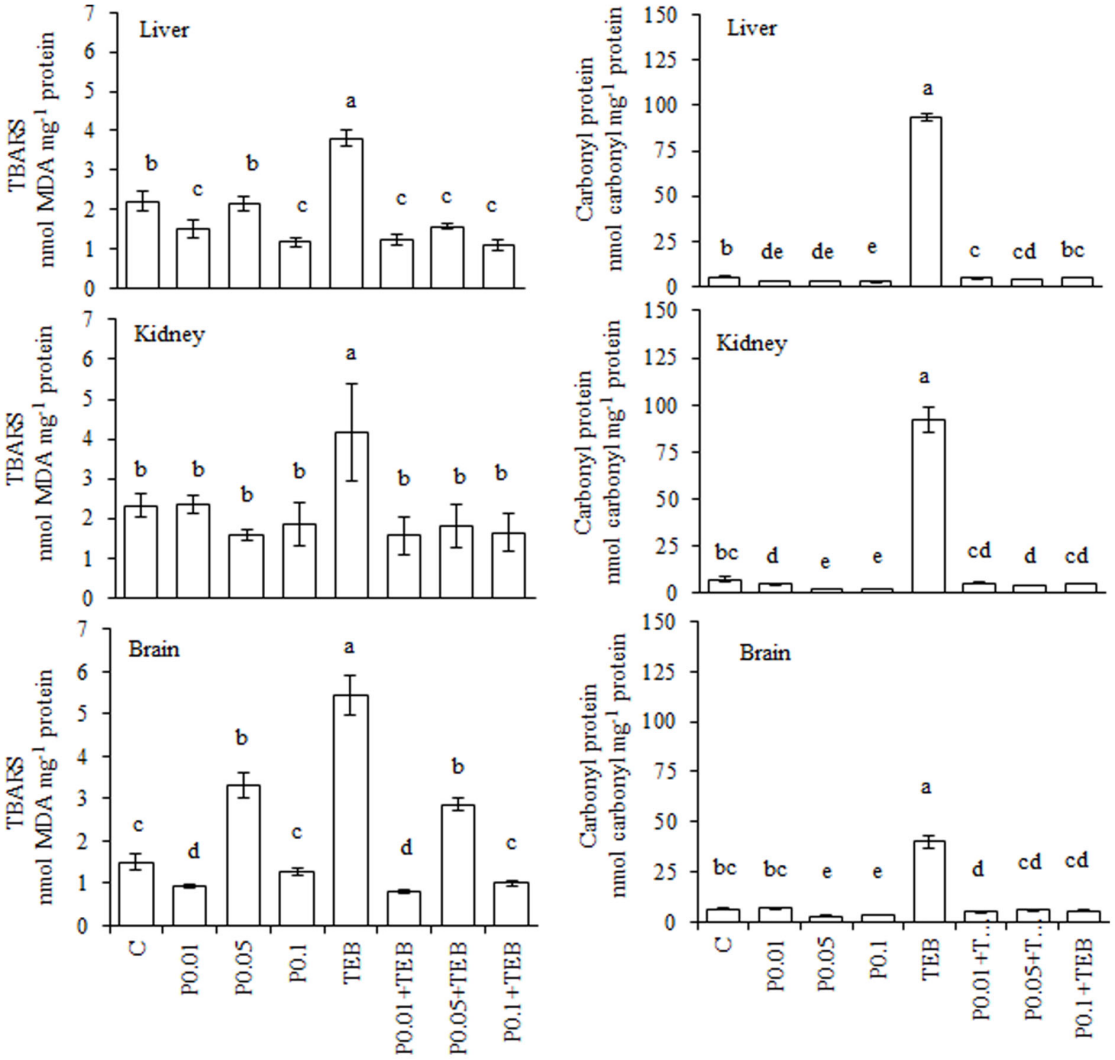
Levels of TBARS (nmol MDA mg^−1^ protein) and protein carbonyls (nmol carbonyl mg^−1^ protein) in *Rhamdia quelen* exposed to tebuconazole (TEB), propolis (P) or a combination of P and TEB. Contaminant-free (C). The different small letters indicate significant differences between the means (ANOVA followed by Tukey's multiple range test). The values represent the means ± SEM; n = 12. p<0.05.

**Figure 6:**
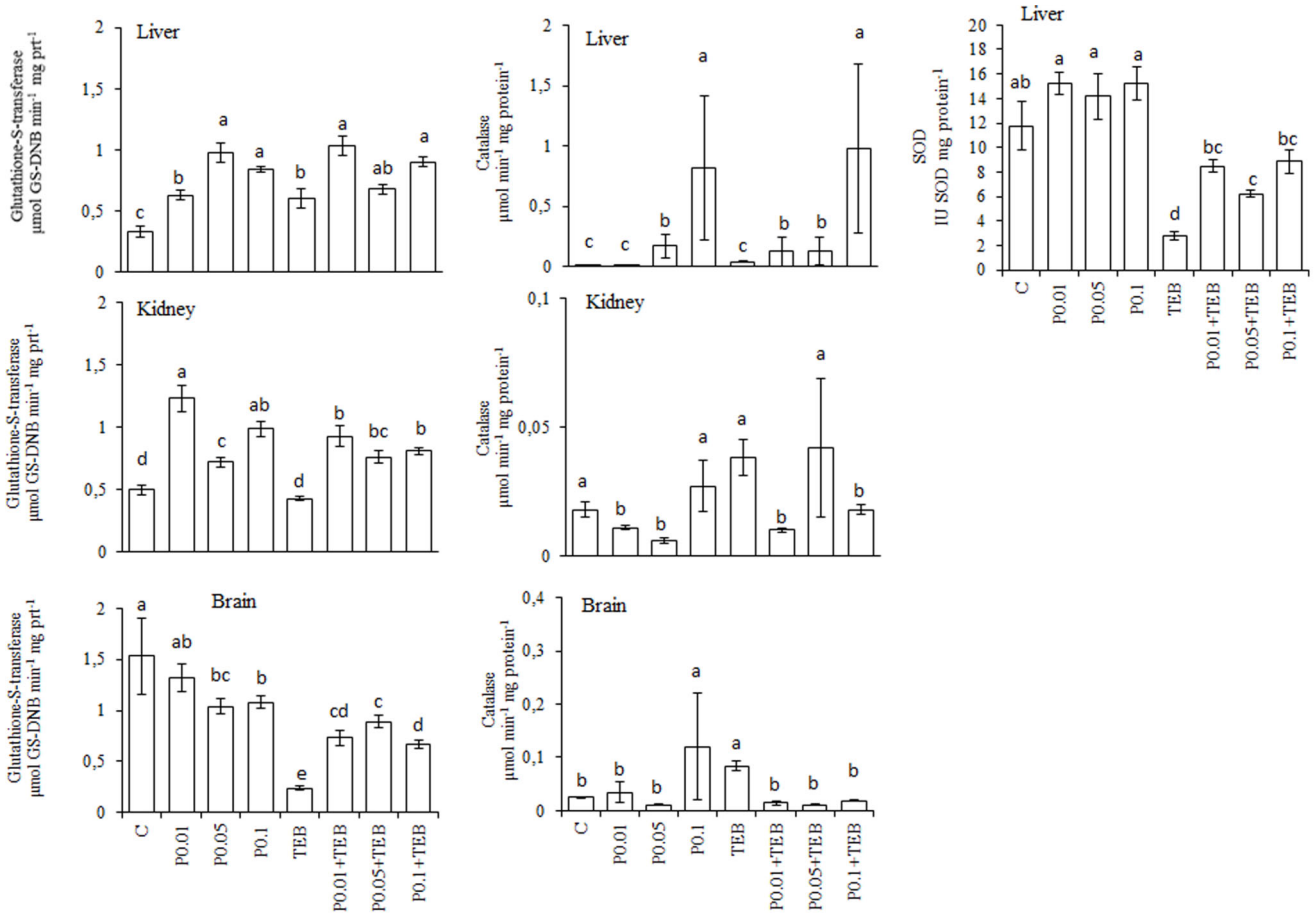
Levels of Glutathione-S-transferase (GST, µmol GS-DNB min^−1^ mg protein^−1^), catalase (CAT, µmol min^−1^ mg protein^−1^) and superoxide dismutase (SOD, IU SOD mg protein^−1^) activities in *Rhamdia quelen* exposed to tebuconazole (TEB), propolis (P) or a combination of P and TEB. Contaminant-free (C). The different small letters after the means indicate significant differences (ANOVA followed by Tukey's multiple range test); p<0.05, n = 12.

TEB treatment increased TBARS generation and protein carbonylation; however, these effects were completely blocked by the P treatments (Fig. 5). The GST, CAT and SOD activities are summarized in Fig. 6. In the liver, the GST and CAT activities were increased in the three P+TEB concentrations relative to the TEB and C fish, whereas SOD activity was higher in the three P+TEB concentrations relative to the TEB-exposed fish. Similarly, GST activity was higher for the three P+TEB treatments relative to the TEB-exposed fish, whereas CAT activity was augmented in TEB and with the P0.05+TEB treatments. In the brain tissue, GST activity was increased in all three P+TEB concentrations relative to the TEB group, and CAT activity in the P+TEB groups was similar to the isolated P groups.

### Bee pollen

**Figure 7:**
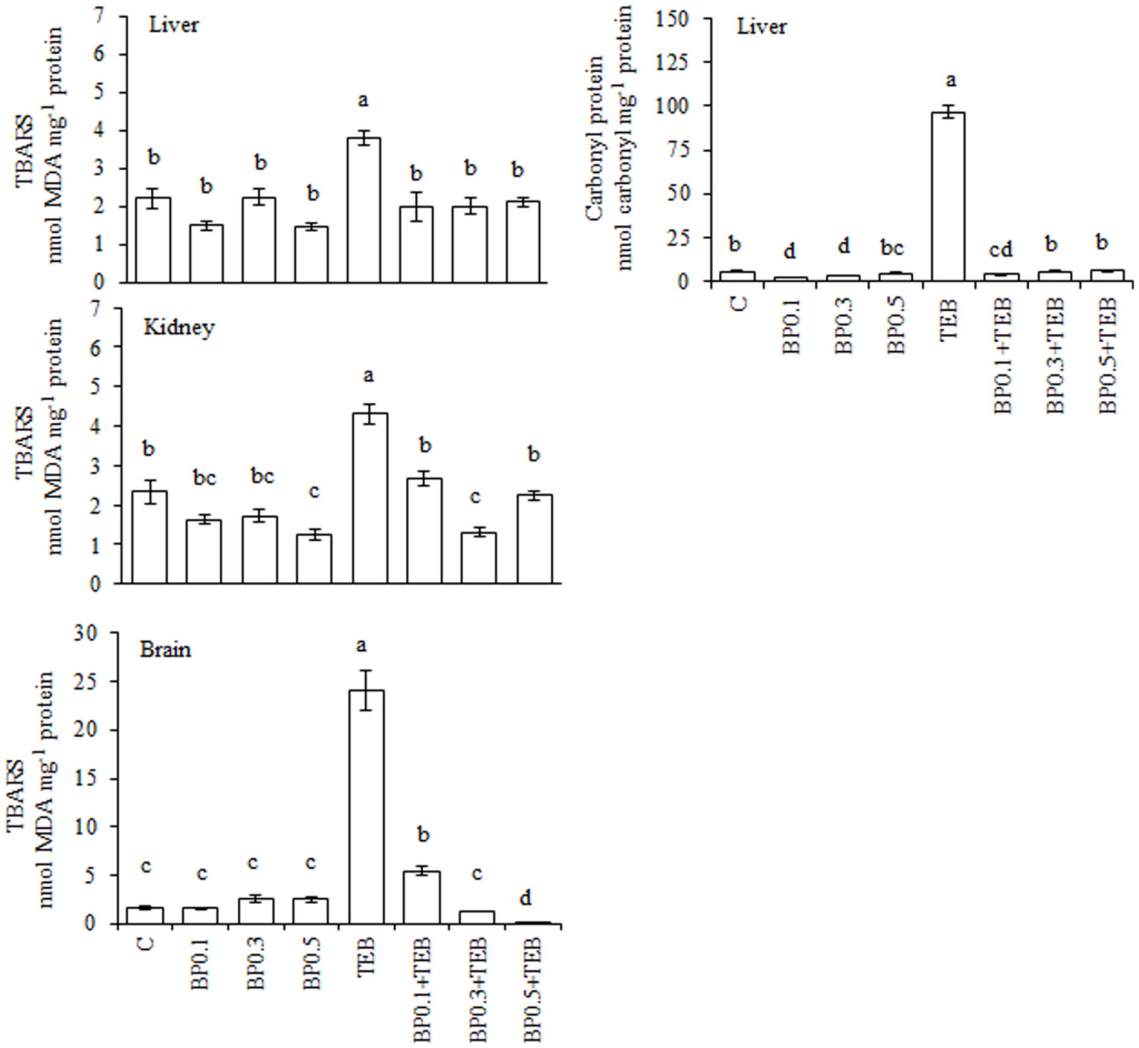
Levels of TBARS (nmol MDA mg^−1^ protein) and protein carbonyls (nmol carbonyl mg^−1^ protein) in *Rhamdia quelen* exposed to tebuconazole (TEB), bee pollen (BP) or a combination of BP and TEB. Contaminant-free (C). The different small letters indicate significant differences between the means (ANOVA followed by Tukey's multiple range test). The values represent the means ± SEM; n = 12. p<0.05.

**Figure 8:**
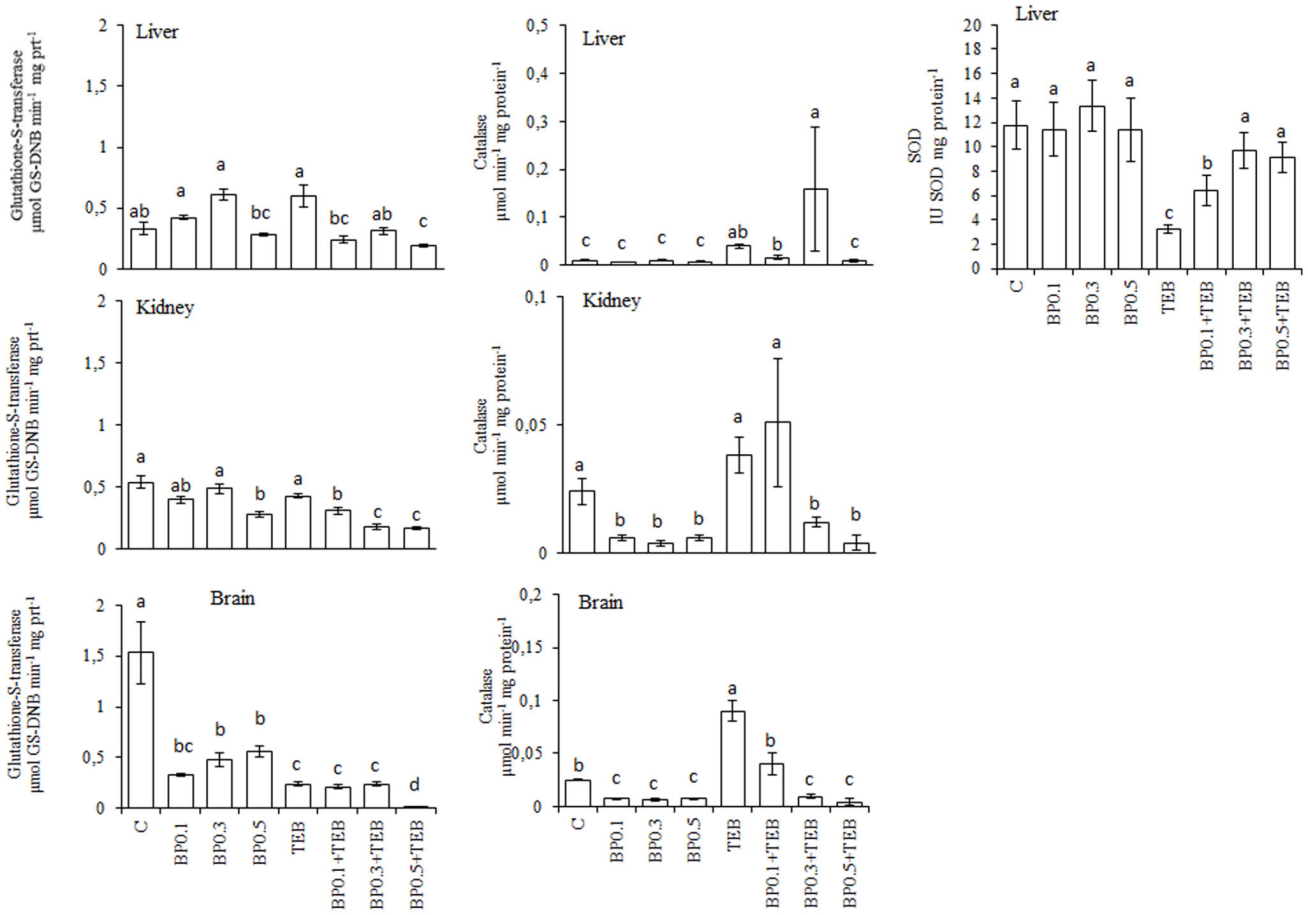
Levels of Glutathione-S-transferase (GST, µmol GS-DNB min^−1^ mg protein^−1^), catalase (CAT, µmol min^−1^ mg protein^−1^) and superoxide dismutase (SOD, IU SOD mg protein^−1^) activities in *Rhamdia quelen* exposed to tebuconazole (TEB), bee pollen (BP) or a combination of BP and TEB. Contaminant-free (C). The different small letters after the means indicate significant differences (ANOVA followed by Tukey's multiple range test); p<0.05, n = 12.

The TEB-induced augmentation of the TBARS generation and protein carbonylation were prevented by BP at every tested concentration (Fig. 7). The anti-oxidant enzymatic activities are depicted in Fig. 8. In the liver, GST activity was increased in the TEB-exposed fish but was similar to the control values for the BP0.1+TEB and BP0.3+TEB treatments. The CAT activity was increased during the TEB and BP0.3+TEB treatments, whereas the SOD activity increased in the three BP+TEB groups. The GST activity decreased in the three BP+TEB groups compared to the TEB-treated and C fish, whereas the CAT was increased in the TEB and BP0.1+TEB groups for kidney tissue. The GST activity in the brain tissue of the BP0.1+TEB and BP0.3+TEB groups was similar to the TEB-exposed fish, whereas the same activity was greatly decreased in the BP0.5+TEB group. The brain tissue exhibited increased CAT activity in TEB-exposed fish, and the activity was similar to the control for BP0.1+TEB.

## Discussion

In this study, we demonstrated that waterborne bee products, including royal jelly, honey, propolis and bee pollen, reversed the oxidative damage induced by TEB exposure. The increased levels of TBARS and protein carbonyls observed in the TEB-exposed fish were prevented or reversed when the TEB-inoculated water was supplemented with various bee products. Our results confirm the previous findings regarding TEB toxicity [16], [17] as well as the antioxidant potential of bee products [11].

Several studies have previously demonstrated the antioxidant properties of bee products, which can be attributed to the high content of phenolic substances. These effects were most likely generated by the high levels of polyphenols in these substances [5]–[9], which can act as antioxidants and free radical scavengers.

LPO is one of the major processes activated in response to xenobiotic-induced oxidative stress. Xenobiotics, such as fungicides, induce LPO in several fish species [31]. LPO can be indirectly assayed by measuring the generation of TBARS, which reflects the intensity of LPO by quantifying malonic dialdehyde, which is one of its main products [24]. The protein carbonyl levels are also a biomarker for xenobiotic exposure in fish. Protein carbonylation causes conformational changes in proteins and reduces their catalytic activity. In addition, carbonylation initiates the degradation of proteins through protease activity [32].

All of the treatments with bee products generated an increase in at least one of the enzymatic antioxidant activities. Therefore, the predominant mechanisms for the reversal of oxidative damage appear to be the augmentation of SOD, CAT and GST enzyme activities.

SOD is the first enzyme to respond to free-radical generation, and it exhibits the most robust response to oxidative stress [33], [1]. This study demonstrated that SOD activity increased in all groups treated for TEB exposure with RJ, H, P and BP compared to TEB-exposed fish. The actions of CAT are primarily catalytic as it promotes the decomposition of hydrogen peroxide to form water and oxygen [34]. CAT protects living organisms from ROS, which are a cause oxidative stress [35]. GST is involved in the detoxification of many xenobiotics and protects tissues from oxidative stress [36]. The GST activity increased in the livers of TEB-exposed fish treated with some of the concentrations of RJ and P. In addition, GST activity was elevated in the livers, kidneys and brains of fish exposed to P. Increased GST activity has been previously observed when *R. quelen* and other fish species were exposed to xenobiotics [37], [38].

We observed a clear interaction between the SOD and CAT activities during the reversal of oxidative damage. The increased SOD activity, which is responsible for catalyzing the conversion of the superoxide anion to H_2_O_2_, might explain the increase in CAT activity, which catalyzes the reduction of H_2_O_2_ to H_2_O and O_2_, in TEB-exposed fish. The decreased CAT activity in “TEB+ bee products” treatments reflects the protective action and/or reversal of damage induced by the bee products. The combined action of both enzymes is a major component of the primary antioxidant defense system [39].

Increasing the enzymatic antioxidant defense network constitutes the most probable mechanism for the bee product-mediated prevention or reversal of oxidative damage caused by TEB. Increases in the enzymatic activity after agrichemical exposure have been previously reported by our laboratory [11], [17].

The proper actions of protective enzymes are essential for the health of a body while combating ROS because ROS are responsible for several deleterious effects, including DNA damage, which ultimately alters and impairs intracellular metabolism and can occasionally cause cell death [40].

The levels of AsA were augmented by RJ, BP and P in all of the studied tissues (data not shown). AsA converts ROS into harmless species, and the derivatives of AsA are unreactive. AsA functions as an antioxidant in vivo [17]); therefore, amplifying the AsA levels may also be an important role in the prevention or reversal of oxidative damage. Previous studies [41], [11] have also found that increases in the AsA contents are an important component of the antioxidant defense system in fish.

We used gross honeybee products instead of isolated or synthetic polyphenols to study, and eventually offer to fish growers, an accessible technology based on products commonly found or produced on a farm. In addition, gross honeybee products are more easily handled and applied in the ponds than isolated and synthetic polyphenols, which require specialized environments and techniques for handling and quantification. Furthermore, the isolated/synthesized polyphenols are difficult and expensive to obtain in the required amounts.

We researched the antioxidant properties of natural products intensively to find economically viable alternatives with low environmental impact; these studies increased the interest in the various compounds contained in bee products as well as *in natura* [10].

In this study, RJ, H, BP and P prevented and/or reversed tissue oxidative damage induced by TEB. Increases in the enzymatic activities of SOD, CAT and GST are the most likely mechanism of action for the protective effects of bee products against tissue oxidation and damage.

Our data opens new possibilities for the usage of honeybee products as nutritional goods for their enhanced antioxidant properties and in aquaculture to protect fish from oxidative damage caused by environmental exposure to pro-oxidant contaminants. In addition, integrated family-based agriculture, where fish, bees and other animals are husbanded together, might facilitate the development of honeybee products for consumption and enhance aquaculture.
